# Medical disease as a cause of maternal mortality:the pre-imminence of cardiovascular pathology

**DOI:** 10.5830/CVJA-2016-018

**Published:** 2016

**Authors:** AO Mocumbi, Karen Sliwa, P Soma-Pillay

**Affiliations:** Instituto Nacional de Saúde and Department of Medicine, Universidade Eduardo Mondlane, Maputo, Moçambique; Hatter Institute for Cardiovascular Research in Africa, and IDM, Department of Medicine, Faculty of Health Sciences, University of Cape Town, South Africa; Soweto Cardiovascular Research Unit, University of the Witwatersrand, Johannesburg; Inter-Cape Heart Group, Medical Research Council South Africa, Cape Town, South Africa; Inter-Cape Heart Group, Medical Research Council South Africa; University of Pretoria and Steve Biko Academic Hospital, Pretoria, South Africa

## Abstract

Maternal mortality ratio in low- to middle-income countries (LMIC) is 14 times higher than in high-income countries. This is partially due to lack of antenatal care, unmet needs for family planning and education, as well as low rates of birth managed by skilled attendants. While direct causes of maternal death such as complications of hypertension, obstetric haemorrhage and sepsis remain the largest cause of maternal death in LMICs, cardiovascular disease emerges as an important contributor to maternal mortality in both developing countries and the developed world, hampering the achievement of the millennium development goal 5, which aimed at reducing by three-quarters the maternal mortality ratio until the end of 2015.

Systematic search for cardiac disease is usually not performed during pregnancy in LMICs despite hypertensive disease, rheumatic heart disease and cardiomyopathies being recognised as major health problems in these settings. New concern has been rising due to both the HIV/AIDS epidemic and the introduction of highly active antiretroviral therapy. Undetected or untreated congenital heart defects, undiagnosed pulmonary hypertension, uncontrolled heart failure and complications of sickle cell disease may also be important challenges. This article discusses issues related to the role of cardiovascular disease in determining a substantial portion of maternal morbidity and mortality. It also presents an algorhitm to be used for suspected and previously known cardiac disease in pregnancy in the context of LIMCs.

## Abstract

The 2010 Millennium Development Goals summit concluded with an action plan to accelerate progress on maternal and child health. The target of the millennium development goal 5 was to reduce by three-quarters the maternal mortality ratio and achieve universal access to reproductive health between 1990 and the end of 2015. A 2013 report by the United Nations states: ‘The maternal mortality ratio dropped by 45 per cent between 1990 and 2013, from 380 to 210 deaths per 100 000 live births. All regions have made progress but accelerated interventions are required in order to meet target.’^1^

The maternal mortality ratio in low- to middle-income countries (LMIC) is 14 times higher than in high-income countries (HIC).^1^ Problems such as lack of antenatal care, the need for family planning and education, and the low rates of birth managed by skilled birth attendants contribute to the high maternal deaths rates in LMICs. Direct causes of maternal death, such as complications of hypertension, obstetric haemorrhage and sepsis remain the largest cause of maternal death in LMICs. Cardiovascular disease however is an important contributor to maternal mortality in both the developing and developed world, justifying a better understanding of its profile and relative burden in both regions.

## Cardiac disease and maternity in high-income countries

**Fig. 1.**
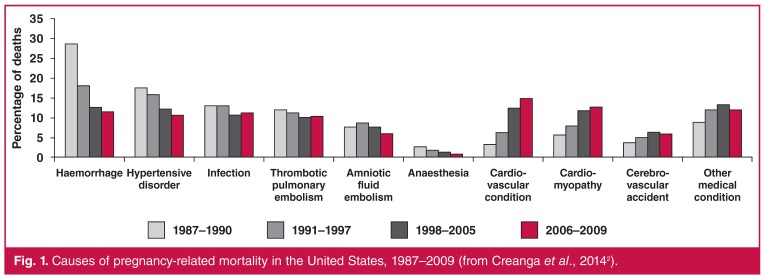
Causes of pregnancy-related mortality in the United States, 1987–2009 (from Creanga et al., 2014^2^).

HIC such as the United States of America and the United Kingdom have reported decreases in direct causes of maternal mortality, but deaths due to cardiovascular disease have remained unchanged or are increasing.^2^,^3^ Considering the surveillance period in the United States (2006–2009), cardiovascular conditions accounted for a third of all pregnancy-related deaths (Fig. 1), while cardiac disease is currently the leading cause of maternal mortality in the United Kingdom.^3^ The most common causes of maternal death in the United Kingdom for the period 2006–2008 were sudden adult death syndrome, peripartum cardiomyopathy, aortic dissection and myocardial infarction.^4^ There were no deaths due to rheumatic heart disease and a decrease in deaths due to congenital heart disease. Lifestyle factors such as advanced maternal age, obesity and smoking were important contributors to maternal mortality.

## Sudden adult death syndrome (SADS)

SADS is defined as sudden death in an adult where no cause is identified. It is believed that obesity, cardiac hypertrophy and severe atherosclerosis can cause arrhythmia and sudden death.^5^ The condition is also associated with the presence of high concentrations of circulating non-esterified fatty acids, and women with central obesity are at greater risk than those with a peripheral pattern of obesity. There were 10 maternal deaths due to SADS in the United Kingdom during the period 2006–2008; four mothers were obese (BMI 30–45 kg/m^2^) and seven had an enlarged heart (the median heart weight at autopsy was 390 g).

## Peripartum cardiomyopathy (PPCM)

PPCM is a potentially life-threatening heart disease emerging towards the end of pregnancy or in the first months postpartum in previously healthy women.^6^ In most patients, cardiac function recovers, however, the mortality rate is up to 5–32%, and many patients develop chronic heart failure.^7^ In the acute phase, PPCM manifests as acute heart failure (AHF) and the diagnosis relies on exclusion of other causes of AHF. A novel finding is the discovery that oxidative stress-mediated cleavage of the nursing hormone prolactin into a smaller biologically active sub-fragment (16-kDA prolactin) may be a major factor initiating and driving PPCM.

Treatment recommendations rely on standard acute heart-failure therapy. After the acute phase, in addition to the standard treatment of chronic heart failure, novel disease-specific strategies, such as bromocriptine, should be considered. PPCM in itself is a prothrombotic condition and embolic events leading to strokes are common. Therefore, all patients with an ejection fraction (EF) < 35%, those receiving bromocriptine, and particularly if a thrombus has been visualised on echocardiography, should be anticoagulated with intravenous heparin or low-molecular weight heparin antepartum, and receive warfarin after delivery.

## Ischaemic heart disease during pregnancy

The incidence of fatal ischaemic heart disease (IHD) in pregnancy ranges between 0.48 and 0.76 per 100 000 pregnancies. The most common presenting symptom in pregnancy is chest pain, which is present in 95% of women with IHD.^8^ In a systematic review of IHD in pregnancy, 93% of women who had an acute myocardial infarct (AMI) due to atherosclerosis had risk factors, compared with AMI caused by coronary dissection (43% had risk factors) and thrombus or emboli (68% had risk factors).^8^ Therefore lifestyle factors such as obesity and smoking are important risk factors in pregnancy.

Coronary artery dissection and thromboembolic coronary events are the most common causes of IHD reported in pregnancy.^8^ Diagnosis in pregnancy is based on ECG (ST-segment deviation will be seen in about 89% of cases) and laboratory investigations. In the United Kingdom sub-standard care due to delayed diagnosis occurred in 46% of cases of maternal death. Therefore, a high index of suspicion is needed for IHD in pregnant women who present with chest pain and risk factors.

## Aortic dissection

In the same United Kingdom registry for the 2006–2008 triennium there were seven maternal deaths due to aortic dissection. In most cases, patients presented with severe chest or interscapular pain requiring opiate analgesia, and the diagnosis was delayed as appropriate investigations were not performed.

Hormonal changes and increased haemodynamic stress predisposes to aortic dissection in pregnancy, but the exact mechanism is unclear.^9^ Obesity, multiparity, raised systolic blood pressure, heart conditions and pre-existing connective tissue disorders such as Marfan and Turner syndrome, Ehlers– Danlos type IV, coarctation of the aorta and bicuspid aortic valve increase the risk for aortic dissection. This diagnosis must be considered in the differential diagnosis of pregnant women who present with chest pain, particularly in the presence of systolic hypertension. Appropriate imaging includes computed tomography chest scan, magnetic resonance imaging, as well as transthoracic or transoesophageal echocardiogram.

## Cardiac disease and maternity in the developing world

Heart disease is a common problem in pregnancy in LMICs;^10^ it increases the risk of morbi-mortality in these women and, as in HICs, seems to be the leading non-obstetric cause of maternal death. Similarly to the situation in HICs, risk factors such as hypertension and diabetes are contributors to maternal morbidity and mortality in LMICs, owing to their prevalence in the general population.^11^-^16^ However, a unique disease profile is found in LMICs due to the existence of poverty-related cardiovascular diseases.

Risk factors such as hypertension, obesity and diabetes are increasingly important, occuring in high numbers in some countries, urban settings and specific sub-populations in sub-Saharan Africa, Asia and Latin America. In Africa, hypertension is most frequently observed in both rural and urban communities, with prevalence rates in young populations ranging from 9.3 to 48.1%.^11^,^12^ Smaller variations were found in India, where the overall prevalence for hypertension was 29.8% (95% confidence interval; range 26.7–33.0%) and significant differences were noted between rural and urban areas.^13^ A metaanalysis of published studies on the prevalence of hypertension in Chinese cities found an average of 21.5%,^14^ while in Iran the prevalence was 41.8% in women.^15^ The Latin American Consortium of Studies in Obesity (LASO), in its study on the major cardiovascular risk factors in Latin America, found that the prevalence of diabetes mellitus, hypertension and low levels of high-density lipoprotein (HDL) cholesterol in Latin American countries were 5, 20.2 and 53.3%, respectively.^16^ In this study, women had a higher prevalence of obesity and lower HDL cholesterol levels than men and, compared to the region’s average, the prevalence of each risk factor tended to be lower in Peru and higher in Chile.

In LMICs, a search for cardiac disease in pregnant women is not performed routinely although hypertensive disease, rheumatic heart disease and cardiomyopathies are recognised as the largest drivers of maternal mortality. Among the cardiomyopathies, peripartum, Chagas disease and endomyocardial fibrosis present specific challenges due to their poor prognosis and high prevalence in some geographical areas. Cardiovascular manifestations of HIV/AIDS is a major concern due to the higher prevalence of infection in African women and lower mean age of those affected, compared to men.^17^

Undetected or untreated congenital heart defects continue to be diagnosed during pregnancy, which is witness to the fact that advances in paediatric cardiology and cardiac surgery over the last decades have benefited disproportionately women from developed countries, compared to those living in the developing world. In fact, pulmonary hypertension and severe heart failure complicating pregnancy are a common presentation when surviving girls reach their reproductive years. In specific endemic 3regions, sickle cell disease and other haemoglobinopathies may constitute important challenges by increasing the risk of thromboembolic complications due to the hypercoagulability caused by enhanced platelet function, activation of the coagulation cascade and impaired fibrinolysis in pregnant women.^18^

## Current data on morbidity and mortality rates

Data on mortality rates of pregnant women with cardiac disease vary widely among LMICs, as data from hospitalbased studies with variable designs and different geographical contexts suggest. Hypertension (pregnancy in hypertensive women and hypertension aggravated by pregnancy) is probably the single most important cardiovascular risk factor linked to adverse maternal and neonatal outcomes in LMICs.^19^ Regarding non-obstetric (indirect) causes, a retrospective analysis on 144 pregnancies in women with cardiac disease who delivered in a single centre in Turkey showed that rheumatic (87.5%) and congenital heart disease (12.5%) were the only causes of disease, with 44.4% of patients presenting in New York Heart Association classes II–IV. Although there was no maternal mortality, morbidity was observed in 16 (11.1%) cases, strongly related to the severity of cardiac disease.^20^ In India, cardiac causes were responsible for 27 of the 277 (9.75%) maternal deaths that underwent a pathological autopsy in a tertiary healthcare centre.^21^ Data from Iran revealed a maternal mortality rate of 4.0% (*n* = 8), with pregnant women with congenital heart disease experiencing higher mortality rates.^22^

Although data from Africa are scarce, maternal morbidity and mortality related to heart disease may reach unacceptably high rates. A systematic review of pre-existing cardiac disease inpregnant women in South Africa found seven studies where the prevalence of heart disease ranged from 123 to 943 per 100 000 deliveries, with a median prevalence of 616 per 100 000.^23^ In this African country, maternal mortality has quadrupled over the last decade, being responsible for 41% of the indirect causes of death; 77% of cardiac deaths occurred in women who attended antenatal clinics, showing major gaps in care and loss of opportunities to diagnose and adequately manage when women contact health services.^24^ Postmortem autopsy findings from cases of maternal death at a tertiary hospital in Nigeria over a five-year period revealed that 84 cases (28.6%) of maternal deaths were due to non-obstetric causes, with 20.8% of them being linked to pre-existing hypertension.^25^

Sliwa *et al.* studied 225 women presenting to a single tertiary care centre in South Africa with cardiovascular disease in pregnancy or within six months’ postpartum, showing that 54% of pregnant women presented to specialised care for the first time with a gestational age over 24 weeks.^26^ This study also showed that women present at late stages of disease, since only 73 (32.4%) were in World Health Organisation (WHO) class I. The most common problems in the 152 women in WHO class II–IV were congenital heart disease (32%), cardiomyopathy (27%) and rheumatic heart disease (26%). Maternal mortality rate within the six-month postpartum follow-up period was 9/152 (5.92%) with all deaths occurring in symptomatic women (WHO class III or IV risk group). The main diagnoses leading to death were familial and peripartum cardiomyopathy (*n* = 7) and prosthetic valve complications (*n* = 2). Interestingly, eight out of nine deaths occurred outside the 42-day maternal mortality report period, meaning that they were not considered in the statistics as maternal deaths.

In LMICs, rheumatic heart disease contributes to 30% of the cardiovascular disease seen in pregnancy and remains an important determinant of morbidity and mortality.^27^-^29^ Rheumatic valvular lesions were the commonest abnormalities found in South Africa, where the most frequent complications were pulmonary oedema, thromboembolism and major bleeding related to warfarin use.^26^ In the global prospective registry of rheumatic heart disease (REMEDY), which enrolled 3 343 patients presenting at 25 hospitals in 12 African countries, India and Yemen, young females were highly represented (median age 28 years, females 66.2%) and had a higher prevalence of major cardiovascular complications.^30^

The participating countries were grouped into three income categories according to 2011 World Bank definitions: low-income countries (Ethiopia, Kenya, Malawi, Rwanda, Uganda and Zambia), lower-middle-income countries (Egypt, India, Mozambique, Nigeria, Sudan and Yemen), and uppermiddle- income countries (Namibia and South Africa). There was no difference in the predominance of females in the three groups: 728/1 110 (65.8%) 867/1 370 (63%) and 616/863 (71.3%), respectively. However, a statistically significant difference was found in the proportion of women in child-bearing years between the three groups of countries (86.5% in low-income countries, 90.3% in LMIC and 66.9% in upper-middle-income countries; *p* < 0.01). Among 1 825 women of childbearing age (12–51 years), only 65 (3.6%) were on contraception, reflecting the poor provision of family planning and pre-pregnancy advice for women with heart disease that occurs in many regions of the world.^31^,^32^

## Socio-demographic and health systems issues

In LMICs, delays in enrolment for antenatal care and lack of adequate healthcare hamper the recognition of life-threatening conditions and the control of preventable factors that lead to cardiac decompensation during pregnancy. Low capacity for diagnosis at peripheral levels of the health systems and lack of awareness of the risks related to pregnancy result in few pregnant women being identified as having heart disease, therefore determining inadequate management and considerable impact on maternal and foetal outcome. On the other hand, acute and chronic complications and common endemic diseases may also further contribute to increase the risk of pregnant women dying during pregnancy, such as the case with cardiac tuberculosis, schistomosmiasis and syphilis.^33^

**Fig. 2.**
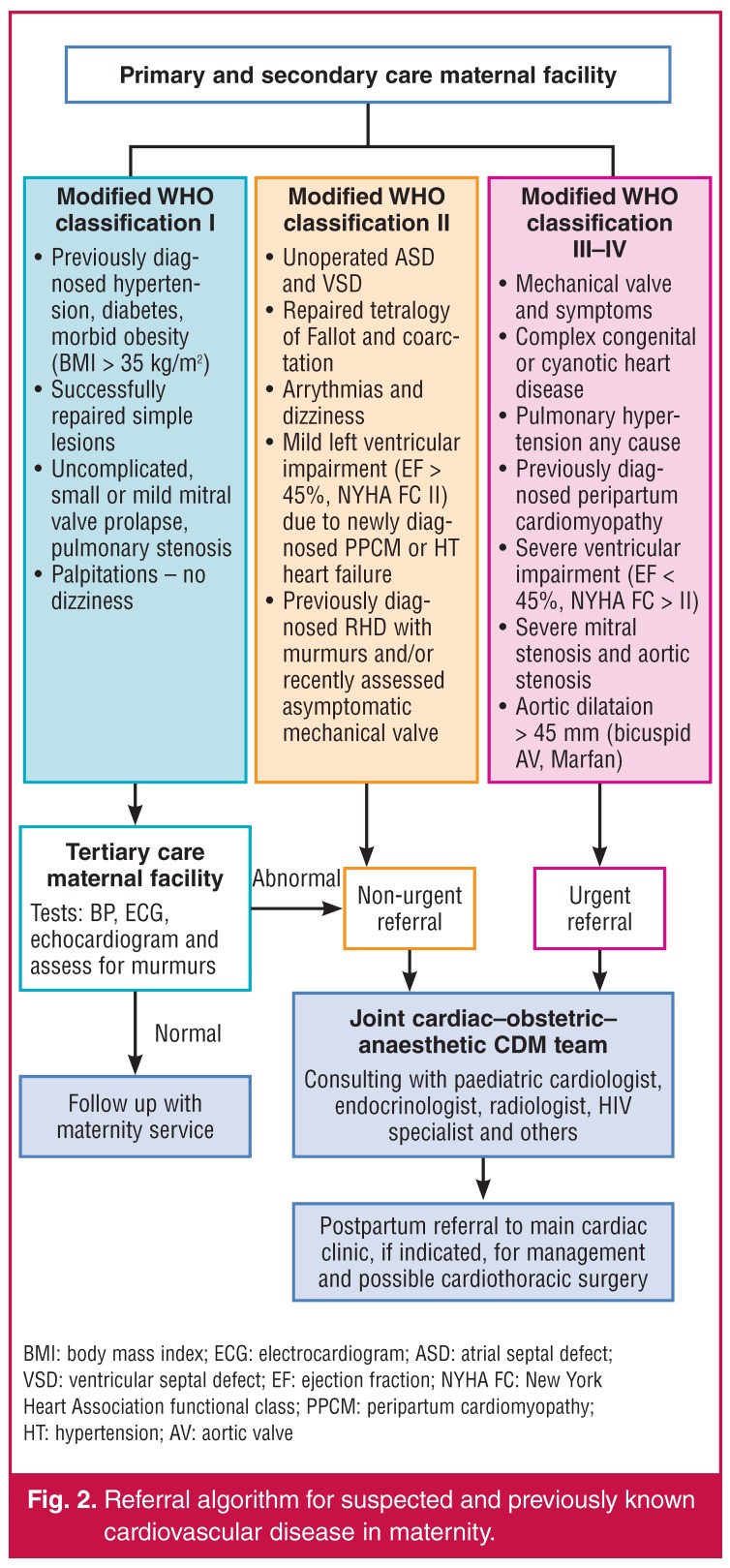
Referral algorithm for suspected and previously known cardiovascular disease in maternity.

For the identification and management of pregnant patients with cardiovascular disease, we would therefore recommend that (1) all pregnant women should be screened at booking for underlying medical or surgical conditions; (2) women with known or recently detected cardiovascular disease should undergo risk assessement based on an algorithm (Fig. 2); (3) women presenting with difficulty in breathing, systolic blood pressure of < 100 mmHg, heart rate > 120 beats per minute or appearing cyanotic, need to be transferred by ambulance to a tertiary centre within 24 hours; those presenting with signs of fluid overload should receive a bolus of lasix 40 mg IV and oxygen per face mask prior to transfer; (4) clinicians should have a low threshold for investigating pregnant or recently delivered women (up to six months postpartum), especially those with cardiovascular risk factors (hypertension, diabetes), suspected rheumatic heart disease or with symptoms such as shortness of breath or chest pain; appropriate investigations include ECG, chest X-ray, echocardiogram and CT pulmonary angiography; (5) certain patients with high-risk cardiovascular disease may need careful monitoring for up to one year postpartum due to the high risk of developing heart failure, serious arrhythmia and embolic events.

Socio-economic and demographic factors such as persistently high fertility rates, social pressure to conceive, insufficient access to contraceptive methods, as well as social or familial ostracism towards women who use contraception, may further contribute to increasing the risk of death due to cardiac disease, even in women who have been diagnosed.^34^

## Conclusions

Available data on maternal mortality rates reveal the pre-imminence of cardiovascular disease as the most important medical cause of non-obstetric maternal death in both developed and developing countries. Failure to systematically search for cardiac disease in pregnant women has led to late diagnosis and high rates of fatal complications. Therefore active screening for cardiac disease in pregnant women is warranted, if the millennium development goal of reducing the maternal mortality ratio is to be achieved.

In LMICs algorithms for cardiac screening of pregnant women should consider the unique profile of cardiovascular disease, including rheumatic heart disease, cardiomyopathies, HIV/AIDS, haemoglobinopathies and undetected/untreated congenital heart defects. Such active strategies for suspected and previously known cardiac disease in pregnancy are expected to prevent a substantial proportion of maternal morbidity and mortality.

## References

[2] Creanga AA, Berg CJ, KO JY (2014). Maternal mortality and morbidity in the United States: Where are we now?. J Women’s Health.

[3] Nelson-Piercy C (2015). The UK maternal death report.. Obstet Med.

[4] Nelson-Piercy C (2011). Cardiac disease in Centre for Maternal and Child Enquiries (CMACE).. Br J Obstet Gynecol.

[5] Zollner J, Curry R, Johnson M (2013). The contribution of heart disease to maternal mortality.. Curr Opin Obstet Gynecol.

[6] Sliwa K, Hilfiker-Kleiner D, Mebazaa A (2014). EURObservational Research Programme: a worldwide registry on peripartum cardiomyopathy (PPCM) in conjunction with the Heart Failure Association of the European Society of Cardiology Working Group on PPCM.. Eur J Heart Fail.

[7] Hilfiker-Kleiner D, Sliwa K (2014). Pathophysiology and epidemiology of peripartum cardiomyopathy.. Nature Rev Cardiol.

[8] Lameijer H, Kampman MAM, Oudijk MA, Pieper PG (2015). Ischemic heart disease during pregnancy or post-partum: systematic review and case series.. Neth Heart J.

[9] Koul A, Hollander G, Moskovits N (2001). Coronary artery dissection during pregnancy and the postpartum period: two case reports and review.. Catherization Cardiovasc Intervent.

[10] Naidoo P, Desai D, Moodley J (2002). Maternal deaths due to pre-existing cardiac disease. Cardiovasc J Afr.

[11] Hendriks ME, Wit FW, Roos MT, Brewster LM, Akande TM, de Beer IH (2012). Hypertension in sub-Saharan Africa: cross-sectional surveys in four rural and urban communities.. PLoS ONE.

[12] Kayima J, Wanyenze RK, Katamba A, Leontsini E, Nuwaha F (2013). Hypertension awareness, treatment and control in Africa: a systematic review.. BMC Cardiovasc Disorders.

[13] Anchala R, Kannuri NK, Pant H, Khan H, Franco OH, Di Angelantonio E, Prabhakaran D (2014). Hypertension in India: a systematic review and meta-analysis of prevalence, awareness, and control of hypertension.. Hypertens.

[14] Ma YQ, Mei WH, Yin P, Yang XH, Rastegar SK, Yan JD (2013). Prevalence of hypertension in Chinese cities: a meta-analysis of published studies.. PLoS ONE.

[15] Malekzadeh M, Etemadi A, Kamangar F, Khademi H, Golozar A, Islami F (2013). Prevalence, awareness and risk factors of hypertension in a large cohort of Iranian adult population.. J Hypertens.

[16] Miranda JJ, Herrera VM, Chirinos JA, Gómez LF, Perel P, Pichardo R (2013). Major cardiovascular risk factors in Latin America: A comparison with the United States. The Latin American Consortium of Studies in Obesity (LASO).. PLoS ONE.

[17] Sliwa K, Carrington MJ, Becker A (2012). Contribution of the human immunodeficiency virus/acquired immunodeficiency syndrome epidemic to de novo presentations of heart disease in the Heart of Soweto Study cohort.. Eur Heart J.

[18] Naik RP, Streiff MB, Lanzkron S (2013). Sickle cell disease and venous thromboembolism: what the anticoagulation expert needs to know.. J Thromb Thrombol.

[19] Paily VP, Ambujam K, Rajasekharan Nair V, Thomas B (2014). Confidential review of maternal deaths in Kerala: a country case study. Br J Obstet Gynecol.

[20] Madazli R, Sal V, Cift T, Guralp O, Goymen A (2010). Pregnancy outcomes in women with heart disease.. Arch Gynecol Obstet.

[21] Panchabhai TS, Patil PD, Shah DR, Joshi AS (2009). An autopsy study of maternal mortality: a tertiary healthcare perspective.. J Postgrad Med.

[22] Yaghoubi A, Mirinazhad M (2013). Maternal and neonatal outcomes in pregnant patients with cardiac diseases referred for labour in northwest Iran.. J Pak Med Assoc.

[23] Watkins D, Sebitloane M, Engel M, Mayosi B (2012). The burden of antenatal heart disease in South Africa: a systematic review.. BMC Cardiovasc Disorders.

[25] Dinyain A, Omoniyi-Esan GO, Olaofe OO, Sabageh D, Komolafe AO, Ojo OS (2013). Autopsy-certified maternal mortality at Ile-Ife, Nigeria.. Int J Womens Health.

[26] Sliwa K, Johnson M, Zilla P, Roos-Hesselink J (2015). Management of valvular disease in pregnancy: a global perspective.. Eur Heart J.

[27] Sliwa K, Libhaber E, Elliot C, Momberg Z, Osman A, Zühlke L (2014). Spectrum of cardiac disease in a low resource cohort in South Africa.. Heart.

[28] Diao M, Kane A, Ndiaye MB, Mbaye A, Bodian M, Dia MM (2011). Pregnancy in women with heart disease in sub-Saharan Africa.. Arch Cardiovasc Dis.

[29] Carapetis JR, Steer AC, Mulholland EK, Weber M (2005). The global burden of group A streptococcal diseases.. Lancet Infect Dis.

[30] Zühlke L, Engel ME, Karthikeyan G, Rangarajan S, Mackie P, Cupido B (2015). Characteristics, complications, and gaps in evidence-based interventions in rheumatic heart disease: the Global Rheumatic Heart Disease Registry (the REMEDY study).. Eur Heart J.

[31] Sliwa K, Mocumbi A (2012). Women’s cardiovascular health in Africa.. Heart.

[32] Thorne S, MacGregor A, Nelson-Piercy C (2006). Risks of contraception and pregnancy in heart disease.. Heart.

[33] Mocumbi AO, Ferreira MB (2010). Neglected cardiovascular diseases in Africa: Challenges and opportunities. J Am Coll Cardiol.

[34] Adongo PB, Phillips JF, Kajihara B (1997). Cultural factors constraining the introduction of family planning among the Kassena-Nankana of Northern Ghana.. Soc Sci Med.

